# Phytosterols and Omega 3 Supplementation Exert Novel Regulatory Effects on Metabolic and Inflammatory Pathways: A Proteomic Study

**DOI:** 10.3390/nu9060599

**Published:** 2017-06-13

**Authors:** Carmen Lambert, Judit Cubedo, Teresa Padró, Joan Sánchez-Hernández, Rosa M. Antonijoan, Antonio Perez, Lina Badimon

**Affiliations:** 1 Cardiovascular Science Institute—ICCC IIB-Sant Pau, 08025 Barcelona, Spain; clambert@santpau.cat (C.L.); jcubedo@csic-iccc.org (J.C.); tpadro@csic-iccc.org (T.P.); 2 Ciber CV, 28029 Madrid, Spain; 3 Ciber DEM, 28029 Madrid, Spain; jsanchezh@comb.cat; 4 Endocrinology Department, Hospital Sant Pau, IIB-Sant Pau, 08025 Barcelona, Spain; APerez@santpau.cat; 5 Medicament ResearchCenter (CIM), Hospital Sant Pau, IIB-Sant Pau, 08025 Barcelona, Spain; rantonijoana@santpau.cat; 6 Cardiovascular Research Chair UAB, 08025 Barcelona, Spain

**Keywords:** phytosterols, omega 3, HDL, LDL, inflammation, lipid metabolism

## Abstract

Cardiovascular disease (CVD) remains one of the major causes of death and disability worldwide. In addition to drug treatment, nutritional interventions or supplementations are becoming a health strategy for CVD prevention. Phytosterols (PhyS) are natural components that have been shown to reduce cholesterol levels; while poly-unsaturated fatty acids (PUFA), mainly omega-3 (ω3) fatty acids, have shown to reduce triglyceride levels. Here we aimed to investigate whether the proteins in the main lipoproteins (low density lipoproteins (LDL) and high density lipoproteins (HDL)) as well as proteins in the lipid free plasma fraction (LPDP) were regulated by the intake of PhyS-milk or ω3-milk, in overweight healthy volunteers by a proteomic based systems biology approach. The study was a longitudinal crossover trial, including thirty-two healthy volunteers with body mass index (BMI) 25–35 kg/m^2^ (Clinical Trial: ISRCTN78753338). Basal samples before any intervention and after 4 weeks of intake of PhyS or ω3-milk were analyzed. Proteomic profiling by two dimensional electrophoresis (2-DE) followed by mass spectrometry-(MALDI/TOF), ELISA, Western blot, conventional biochemical analysis, and in-silico bioinformatics were performed. The intake of PhyS-milk did not induce changes in the lipid associated plasma protein fraction, whereas ω3-milk significantly increased apolipoprotein (Apo)- E LDL content (*p* = 0.043) and induced a coordinated increase in several HDL-associated proteins, Apo A–I, lecitin cholesterol acyltransferase (LCAT), paraoxonase-1 (PON-1), Apo D, and Apo L1 (*p* < 0.05 for all). Interestingly, PhyS-milk intake induced a reduction in inflammatory molecules not seen after ω3-milk intake. Serum amyloid P component (SAP) was reduced in the LPDP protein fraction (*p* = 0.001) of subjects taking PhyS-milk and C-C motif chemokine 2 (CCL2)expression detected by reverse transcription polymerase chain reaction (RT-PCR) analysis in white blood cells was significantly reduced (*p* = 0.013). No changes were observed in the lipid-free plasma proteome with ω3-milk. Our study provides novel results and highlights that the PhyS-milk induces attenuation of the pro-inflammatory pathways, whereas ω3-milk induces improvement in lipid metabolic pathways.

## 1. Introduction

Cardiovascular disease (CVD) is a very common pathology that is the main cause of death and disability worldwide. The reduction of CVD risk is one of the major challenges of cardiovascular medicine. 

Nowadays, the incidence of overweight and obesity is increasing, becoming an important risk factor for a number of diseases including atherosclerosis and CVD [1]. Moreover, several studies have demonstrated, and it is generally acknowledged, that lifestyle and nutritional habits are closely associated with the presentation of CVD [2,3,4].

A very common therapeutic strategy for the prevention of CVD is nutritional intervention or supplementation. The cardioprotective effects of poly-unsaturated fatty acids (PUFA) intake have been examined in several studies, which have established that diets enriched in omega 3 poly-unsaturated fatty acids (ω3-PUFA) from plants and fish have an important role in the prevention of CVD [5,6]. Some of the beneficial effects of PUFA rich foods include a reduced susceptibility to suffer from ventricular arrhythmia, antithrombogenic and antioxidant effects, retardation of atherosclerotic plaque growth, improved blood lipid and lipoprotein profile, and also anti-inflammatory and hypotensive effects. PUFA supplemented foods have been shown to reduce triglyceride (TG) levels [7]. Additionally, dietary supplementation with phytosterols (PhyS) has been shown to reduce the risk of CVD and it is a common nutritional strategy to reduce cholesterol levels [8].

Among the different lipid-associated plasma fractions, low-density lipoproteins (LDL) and high-density lipoproteins (HDL) are widely studied. In primary prevention, high levels of LDL-cholesterol (LDL-C) are related with a higher incidence of cardiovascular events in the continuum of CVD, whereas HDL-cholesterol (HDL-C) levels are commonly known as a risk-reducing factor [9]. It is now accepted that the importance of HDL in the progression of CVD, resides on their quality rather than their quantity, highlighting the importance of their composition, structure, and function [10,11,12,13]. Indeed, apolipoprotein A–I (Apo A–I), the major protein component of HDL, has cardiovascular protective properties [14,15]. Also, low levels of apolipoprotein E (Apo E) have been related to hyperlipidemia and atherosclerosis [16]. 

In addition to apolipoproteins, HDL-associated enzymes such as paraoxonase-1 (PON-1) and lecitin cholesterol acyltransferase (LCAT) exert antioxidant and cardioprotective effects [17]. In fact, the association of LCAT, PON-1, and Apo A–I have been shown to increase the time span of HDL protection against LDL oxidation [18]. 

Beside lipid metabolism, another key hallmark in the pathogenesis of atherosclerotic disease is inflammation. One of the main protein families involved in systemic inflammation are pentraxins [19]. Pentraxins are serum proteins with a relatively uncommon pentameric structure, which have a common amino acid domain in the C-terminal region (pentraxin signature), and besides their role in inflammation they also have a role in immunity and homeostasis [20,21]. Depending on the length of the amino acid chain, pentraxins are divided in two subfamilies, long pentraxins and short pentraxins. Members of the short pentraxins include C-reactive protein (CRP) and serum amyloid P component (SAP), which are acute-phase proteins secreted mainly by hepatocytes in response to pro-inflammatory stimuli. In fact, the role of CRP as a risk marker for atherosclerosis has been widely studied [21].

Our group has previously demonstrated that the intake of low fat milk supplemented with PhyS reduces plasma cholesterol levels; whereas ω3 supplementation reduces plasma TG and very-low density lipoprotein (VLDL) cholesterol levels [7]. Additionally, PhyS and ω3 supplementation induce a differential shift in the LDL lipidomic profile [7]. In the present study, we aimed to further characterize and extend PhyS and ω3 induced changes by analyzing the differential proteomic profile of the lipid-associated plasma protein fraction (LDL and HDL) and the soluble protein fraction of plasma (LPDP) in a subgroup of subjects of our previously reported study, in order to broaden our in-depth understanding of the effect of these food supplements and evidence further effects at a molecular level that could provide CVD protection.

## 2. Materials and Methods 

### 2.1. Study Population

**Figure 1:**
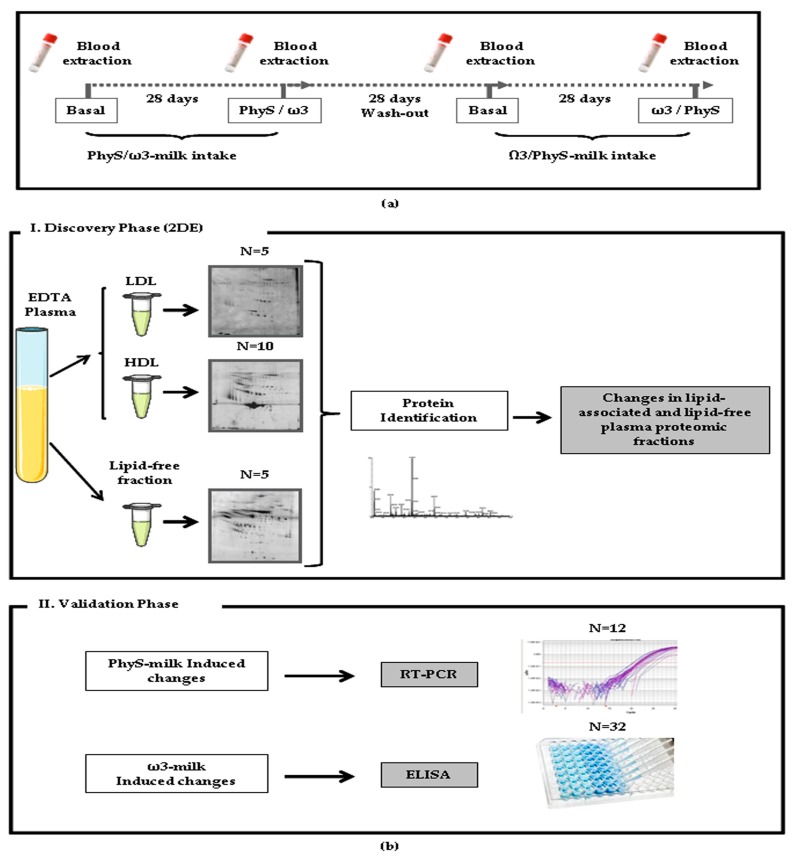
Study design. (**a**) Blood samples were obtained before and after the 28 day milk supplementation treatment period. A 28 day wash-out was made between treatments; (**b**) The study workflow of the present study comprised two phases: (**I**) the discovery phase in which a proteomic approach was used to identify changes in the proteomic profile of the different plasma fractions after the intake of supplemented milk (*N* = 5); and (**II**) the validation phase, where different selected proteins were validated by ELISA (*N* = 32) and inflammatory changes were analyzed by reverse transcription polymerase chain reaction (RT-PCR; *N* = 12).

This is a sub-study of a previously reported double-blinded randomized two-arm longitudinal crossover trial [Clinical Trial: ISRCTN78753338] [7]. All subjects were submitted to two 28-day intervention periods in which volunteers were instructed to consume 250 mL of ω3-supplemented milk (131.25 mg EPA + 243.75 mg DHA/250 mL of milk) or PhyS-supplemented milk (1.6 g of plant sterols/250 mL of milk), separated by a 4 weeks wash-out period (Figure 1a). Both products were prepared by CAPSA Food (Spain) and with no identification of the product administrated. PhyS-milk was commercially available, whereas ω3-milk was prepared for the study. Before the initiation of the intervention, individuals were submitted to a 2 weeks run-in period. During the run-in and wash-out periods, participants received a commercially available plain low-fat milk (without PhyS or ω3), with the same composition to that used for preparing the PhyS- and ω3-enriched milks. These basal samples are used as self-control to avoid variability between volunteers. The trial demonstrated that milk-supplementation with 1.6 g of PhyS significantly reduces LDL-C and milk with 375 mg of ω3 significantly reduces TG levels [7].

Healthy volunteers between the ages of 25 and 70 years (*N* = 32), attending to regular medical controls, were eligible for participation if they had overweight or grade 1 obesity (BMI 25–35 kg/m^2^; Table 1). Subjects were excluded if they reported existing chronic illnesses including cancer, overt hyperlipidemia, diabetes mellitus, hypertension, or heart, liver, or kidney disease. Volunteers were excluded if they were under anti-inflammatory medication or any blood thinning treatment during the study or during the 2-weeks run-in period. Other exclusion criteria were: use of lipid-lowering drugs, β-blockers, or diuretics, history of CVD, lactose intolerance, or being in a weight-loss program. To confirm health status, all subjects underwent a complete physical examination conducted by the physician of the study. Those consuming a PhyS-enriched spread and/or fish oil supplements or with strong aversion to milk derived products were also excluded. Compliance was controlled by telephone and personal interview and with a written formulary on each period. The Human Ethical Review Committee of the Hospital Sant Pau in Barcelona approved the study. Informed written consent was obtained from all participants. Reporting of the study conforms to the STROBE (strengthening the reporting of observational studies in epidemiology)-guidelines.

### 2.2. Study Phases

The study was subdivided in two phases: (1) A discovery phase where the proteomic profile of the LDL fraction (*N* = 5), HDL fraction (*N* = 10) and lipid-free plasma fraction (*N* = 5) was investigated by bi-dimensional electrophoresis, followed by mass spectrometry (MS)identification (Figure 1b-I) in those subjects that showed the highest response in LDL-C and TG plasma levels after the intervention; and (2) a second phase in which the detected changes were validated by complementary methodologies (reverse transcription polymerase chain reaction (RT-PCR) and ELISA; Figure 1b-II).

Twelve hours fasting blood samples were collected on days 1 and 28 (baseline and endpoint of the first treatment period) and on days 56 and 84 (baseline and endpoint of the second treatment period), as previously described [7]. Blood samples were collected without anticoagulant or in EDTA-containing Vacutainer tubes for serum and plasma preparation, respectively. Serum and plasma fractions were separated by centrifugation at 3000× *g* for 20 min at 4 °C and stored at −80 °C until analysis.

### 2.3. Sample Preparation

Lipoprotein fractions were prepared in KBr density gradients (1.019–1.063 for LDL and 1.063–1.210 for HDL) [7,22,23,24]. Lipoprotein purity was routinely analyzed by electrophoresis (2 µL sample) in agarose gels using a commercial assay (SAS-MX Lipo 10 kit; Helena Biosciences, Gateshead, UK), as described by the providers. In addition, LDL purity was checked by analyzing the LDL profile in samples of randomly selected subjects (one subject per ultracentrifugation batch) by chromatography analysis by microgel filtration using a Superose 6 PC 3.2/30 column and an Agilent 1200 HPLC system, as previously described. Briefly, 10 µL of undiluted LDL sample fraction were loaded in the system and run with a constant flow of 100 µL/min. The retention time for the LDL fraction (130 min) was compared with that for HDL (134 min). The method of Karlsson et al. [25] was used for LDL protein mapping, with minor modifications as previously described [26]. Briefly, 1 mL of LDLs (1 g/L Apo B) was delipidated by mixing with 14 mL of ice-cold tributylphosphate:acetone:methanol (1:12:1) and incubating for 90 min at −20 °C, followed by centrifugation at 2800× *g* for 15 min. Protein pellets were washed sequentially with 1 mL of tributyl phosphate, acetone, and methanol, and then air dried. Precipitates were boiled in solution containing 0.325 M DTT, 4% chaps, and 0.045 M Tris for 3 min, cooled at room temperature, diluted (1:15) in urea/thiourea/chaps solution, and incubated at 35 °C for 15 min. For proteomic studies, HDL samples were prepared as previously described [22,23,24,26] by precipitation with pure ice-cold acetone and were solubilized in a urea/thiourea buffer (7 M urea, 2 M thiourea, 2% CHAPS).

In order to analyze only the soluble proteins present in plasma, microparticles were removed from LPDP samples by centrifugation at 25,000× *g* for 45 min at room temperature. For proteomic studies, LPDP was sonicated in ice and filtrated (0.22 µm) by centrifugation to avoid the presence of impurities. The 14 most abundant plasma proteins were depleted by using a specific affinity cartridge as reported by the providers (Multiple Affinity Removal Spin Cartridge, Agilent Technologies, Santa Clara, CA, USA). LPDP fractions were concentrated and de-salted by centrifugation with 5 kDa cutoff filter devices and sample buffer was exchange to a urea containing buffer (8 M urea, 2% CHAPS). Protein concentration was measured with 2D-Quant Kit (GE Healthcare, Little Chalfont, UK). All processed samples were stored at −80 °C until use.

### 2.4. Differential Proteomic Profiling Analysis

#### 2.4.1. Two-Dimensional Gel Electrophoresis (2-DE)

A protein load of 100 μg (analytical gels) and 300 μg (preparative gels) of the urea/thiourea/chaps LDL or HDL extracts or of the urea/chaps LPDP extracts was applied to 17-cm dry strips (pH 4–7 linear range; BioRad, Hercules, CA, USA). The second dimension was resolved in 10–12% SDS-PAGE for LPDP and LDL or HDL samples, respectively. Gels were developed by fluorescent staining (Flamingo; BioRad, Hercules, CA, USA). For each independent experiment, 2-DE for protein extracts from baseline and post-PhyS/ω3-milk intake were processed in parallel to guarantee a maximum of comparability. Analysis for differences in protein extracts was performed with the PD-Quest 8.0 (BioRad, Hercules, CA, USA). Each spot was assigned a relative value (AU) that corresponds to the single spot volume compared to the volume of all spots in the gel, following background extraction and normalization between gels, as previously reported [22].

#### 2.4.2. Mass Spectrometry Analysis

Proteins were identified after in-gel tryptic digestion and extraction of peptides from the gel pieces by matrix-assisted laser desorption/ionization time-of-flight (MALDI-TOF) using an AutoFlex III Smart beam MALDI-TOF/TOF (BrukerDaltonics, Billerica, MA, USA), as previously described [23,26]. Samples were applied to Prespotted Anchor Chip plates (BrukerDaltonics, Billerica, MA, USA) surrounding the calibrants provided on the plates. Spectra were acquired with flex control on reflector mode, (mass range: 850–4000 *m*/*z*, reflector 1:21.06 kV; reflector 2:9.77 kV; ion source 1 voltage: 19 kV; ion source 2:16.5 kV; detection gain: 2.37×) with an average of 3500 added shots at a frequency of 200 Hz. Each sample was processed with FlexAnalysis (version 3.0, BrukerDaltonics, Billerica, MA, USA) considering a signal-to-noise ratio over 3, applying statistical calibration and eliminating background peaks. For identification, peaks between 850 and 1000 *m*/*z* were not considered. After processing, spectra were sent to the interface BioTools (version 3.2, BrukerDaltonics, Billerica, MA, USA) and a MASCOT server search on the Swiss-Prot 57.15 database was done (Taxonomy: Homo Sapiens, Mass Tolerance 50 to 100, up to 2 trypsin miss cleavages, Global Modification: Carbamidomethyl (C), Variable Modification: Oxidation (M)). Identified proteins were accepted when a mascot score higher than 50 was obtained by peptide mass fingerprint and confirmed by peptide fragmentation working in the reflection mode.

### 2.5. Western Blot Analysis

Protein extracts were resolved by 1-DE under reducing conditions and electrotransferred to nitrocellulose membranes in semi-dry conditions (Trans-Blot Turbo system; BioRad, Hercules, CA, USA). Serum amyloid P (SAP) detection was performed using a mouse monoclonal antibody against total SAP (ab27313, 1:200 dilution, abcam, Cambridge, UK). Band detection was performed using a chemiluminiscent substrate dye (Luminata Forte Western HRP Substrate, Merck Millipore, Billerica, MA, USA) and a molecular imager ChemiDoc XRS System, Universal Hood II (BioRad, Hercules, CA, USA). Band quantification was performed with Image Lab 4.0 software (BioRad Laboratories, Hercules, CA, USA). Protein load was normalized with total protein staining, as previously described [23].

### 2.6. Quantification of Total Protein Systemic Levels

Total Apo E, Apo A–I, and LCAT levels in the serum samples from the basal condition and after the intake of ω3-milk were measured by using a commercial sandwich-based ELISA kit in the whole cohort (*N* = 32; Table 1). The detection limit of the assays were: 1.5 ng/mL for Apo E (ELH-Apo E; Human Apo E ELISA Kit; RayBiotech, Norcross, GA, USA); 0.7 µg/mL for Apo A–I (EA5201-1; Human Apo A–I ELISA Kit; AssayPro, St. Charles, MO, USA); and 0.27 ng/mL for LCAT (RD191122200R; LCAT ELISA Kit; BioVendor, Brno-Řečkovice a MokráHora, Czech Republic).

### 2.7. Gene Expression Analyses

RNA was extracted from total blood samples in a randomly selected group of volunteers using a commercial kit (PreAnalytiX, PAX gene, Quiagen/BD Company, Hilden, Germany) and DNA synthesis was performed using a commercial RT First Strand kit. PAX gene tubes avoid RNA degradation during the transport and storage of blood samples. mRNA levels were analyzed by real-time PCR using TaqMan gene expression assays and the Prism 7900HT Sequence detection System (all from Thermo Fisher Scientific, Waltham, MA, USA) according to the manufacturer’s instruction. All expression analyses were normalized with glyceraldehyde-3-phosphate dehydrogenase (GAPDH). 

### 2.8. Bioinformatic Analysis

The statistically significant neural network and canonical pathway in which the identified proteins were involved were generated through the use of IPA (Ingenuity System, www.ingenuity.com).

#### 2.8.1. Functional Analysis of a Network

The functional analysis of a network was used to identify the biological functions and/or diseases that were most significant to the molecules in the network. The network molecules associated with biological functions and/or diseases in the Ingenuity Knowledge Base were considered for the analysis. Right-tailed Fisher’s exact test was used to calculate a *p*-value determining the probability that each biological function and/or disease assigned to that network is due to chance alone.

#### 2.8.2. Canonical Pathway Analysis

Canonical pathway analysis was used to identify the pathways from the IPA library that were most significant to the data set. The significance of the association between the data set and the canonical pathway was measured in two ways: (1) a ratio of the number of molecules from the data set that maps to the pathway divided by the total number of molecules that maps to the canonical pathway is displayed; and (2) Fisher’s exact test was used to calculate a *p*-value determining the probability that the association between the genes in the data set and the canonical pathway is explained by chance alone.

### 2.9. Statistical Analysis

Data are expressed as mean and standard error unless stated. N indicates the number of subjects tested. Statistical analysis was performed with Stat View 5.0.1 software (SAS Institute, Cary, NC, USA). Differences between the basal condition and after 4 weeks of intake of supplemented milk were tested using repeated measurements ANOVA analysis. A *p*-value ≤ 0.05 was considered significant.

## 3. Results

### 3.1. Four Weeks ω3-Milk Intake Induces Changes in the Proteomic Profile of the Lipoprotein Plasma Fraction

**Figure 2:**
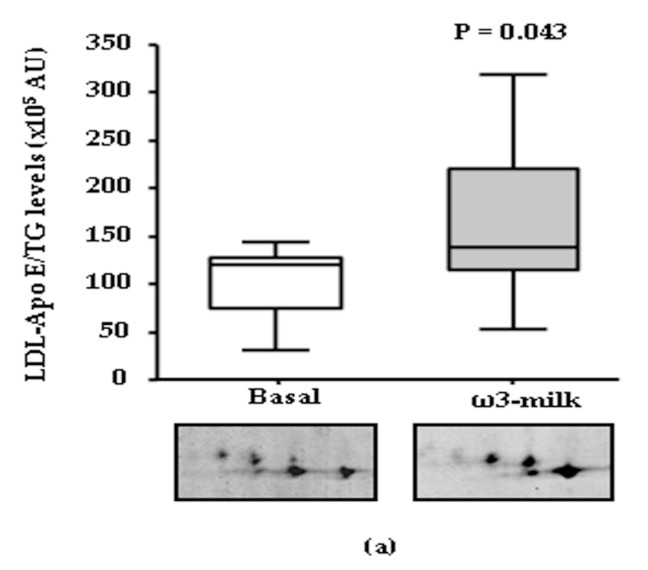

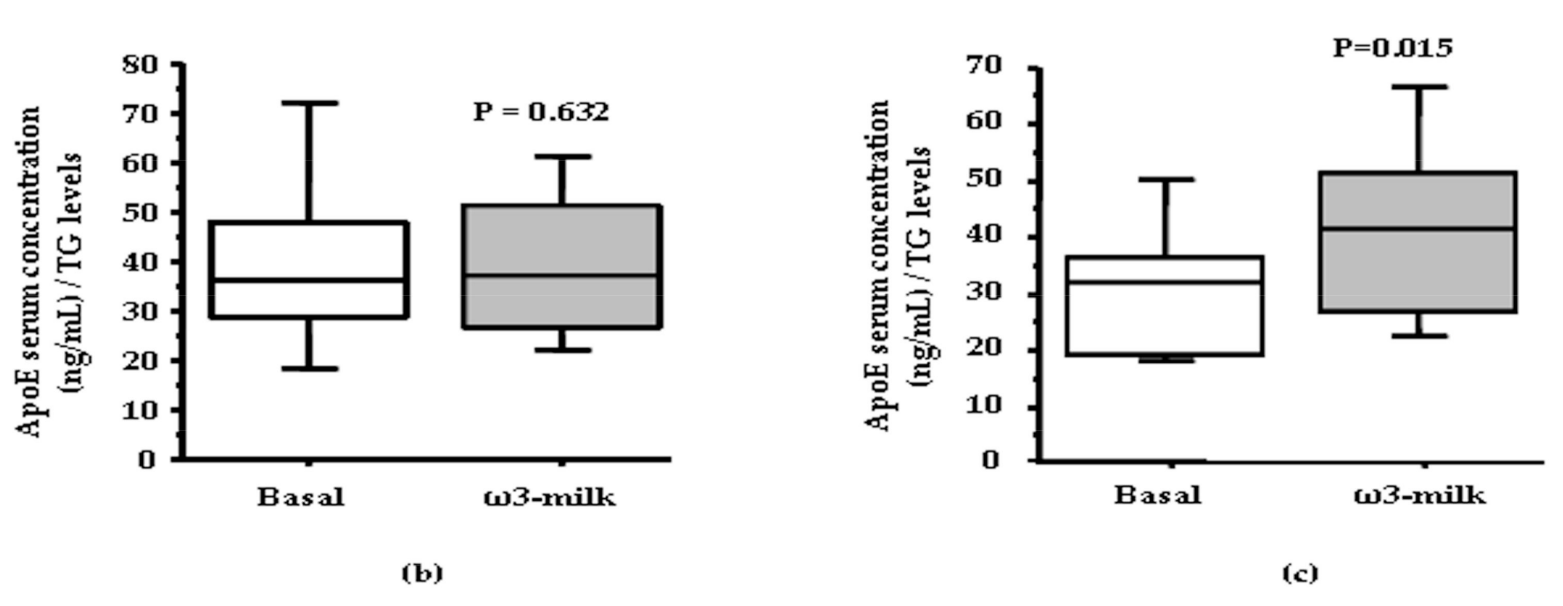
Changes in the apolipoprotein (Apo) E profile. (**a**) Box-Plot diagram and representative 2-DE images showing the low density lipoprotein (LDL)-associated Apo E proteomic profile before and after the intake of ω3-milk. A significant increase of Apo E levels (*p* = 0.043) is observed. Box-Plot diagrams showing serum Apo E levels (ng/mL) in basal conditions and after 4-weeks intervention with ω3-milk, measured by a commercial ELISA. No change was observed when all the volunteers were analyzed ((**b**); *N* = 32; *p* = 0.105). Apo E concentration of subjects with reduced triglyceride (TG) levels showed a significant increase after ω3-milk intake ((**c**); *N* = 11; *p* = 0.015).

The intake of PhyS-milk did not induce any significant change in the LDL proteome (Supplementary Table S1). Only a trend to increased apolipoprotein A-IV (Apo A-IV) levels was observed (*p* = 0.080). On the contrary, the intake of ω3-milk (Supplementary Table S2) induced a 1.5-fold significant increase in the Apo E content in LDL (*p* = 0.043; Figure 2a). To analyze if the observed changes in Apo E in the LDL fraction were also found in total serum Apo E levels, a commercial ELISA was run in serum samples of the whole cohort of individuals (*N* = 32). No changes were observed in total Apo E serum levels (Figure 2b). However, a significant increase in Apo E serum levels was observed (*p* = 0.015; Figure 2c) only when individuals (*N* = 11) that showed a reduction in TG plasma levels (30.3% mean decrease) after the intake of ω3-milk were examined.

**Figure 3:**
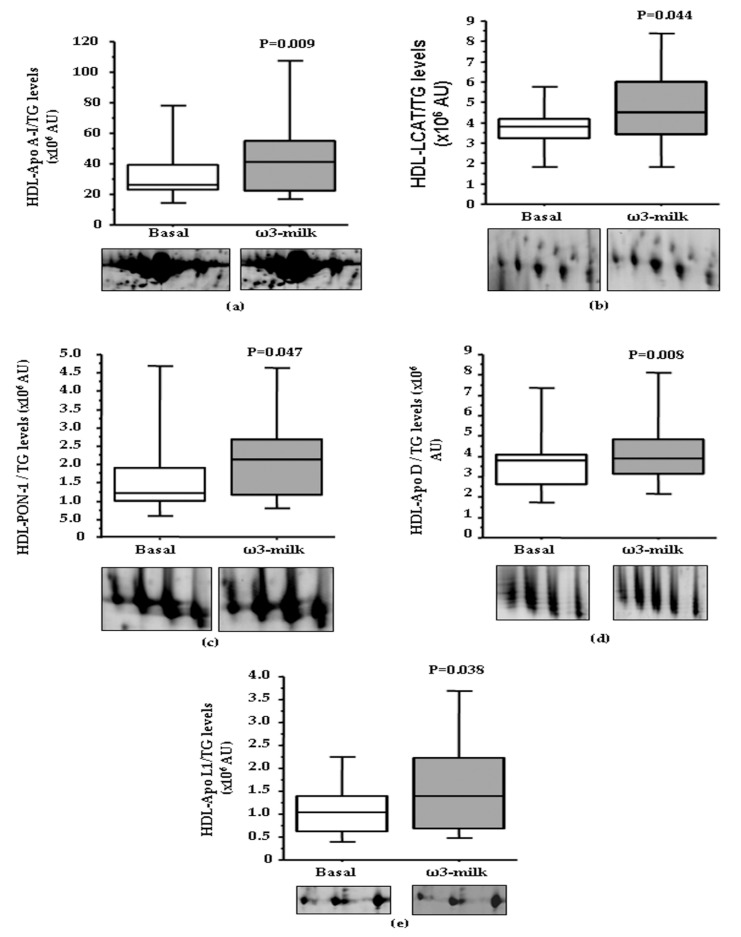
Impact of the intake of ω3-milk on the high density lipoprotein (HDL) profile. Box-Plots and 2-DE representative images showing the significant changes induced in HDL proteins by ω3-milk: (**a**) Apo A–I (*p* = 0.009); (**b**) lecitin cholesterol acyltransferase (LCAT) (*p* = 0.044); (**c**) paraoxonase-1 (PON-1) (*p* = 0.047); (**d**) Apo D (*p* = 0.008); and (**e**) Apo L1 (*p* = 0.038).

The intake of ω3-milk induced a coordinated increase in the content of several key HDL protein components. Among the observed protein changes, ω3-milk induced a significant increase of the main HDL protein component, Apo A–I (*p* = 0.009; Figure 3a). Furthermore, there was a coordinated increase of two important enzymes involved in HDL metabolism, LCAT (*p* = 0.044; Figure 3b) and PON-1 (*p* = 0.047; Figure 3c). In addition, ω3-milk also increased the HDL content of Apo D (*p* = 0.008; Figure 3d) and Apo L1 (*p* = 0.038; Figure 3e). In silicobioinformatic analysis revealed that all the ω3-milk induced changes in the plasma proteome were related to HDL metabolism-related pathways (Supplementary Figure S1).

**Figure 4:**
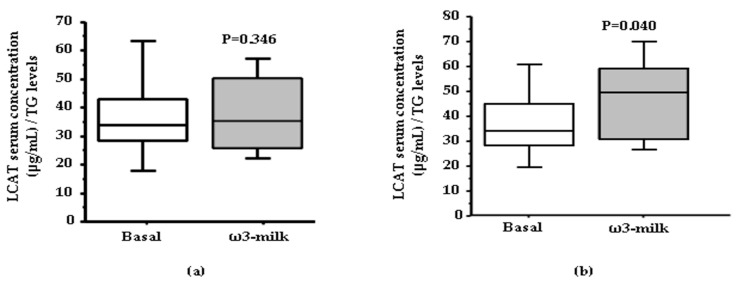
Changes on lecitin cholesterol acyltransferase (LCAT) serum levels after ω3-milk intake. Box-Plot diagrams showing LCAT concentration (µg/mL) in basal conditions and after the intake of ω3-milk, measured by a commercial ELISA. (**a**) No change was observed when all the volunteers were analyzed (*N* = 32; *p* = 0.346). (**b**) There was a significant increase in LCAT serum levels in subjects that showed a reduction in TG levels after ω3-milk intake (*N* = 11; *p* = 0.0397).

In order to analyze if the observed HDL-associated protein changes were translated into changes in total serum protein levels, Apo A–I and LCAT serum levels were measured with commercial ELISAs in the whole study population. This analysis revealed a lack of differences in both Apo A–I and LCAT (Figure 4a) total serum levels after ω3-milk intake when compared to basal levels. However, and as observed with Apo E, if only those subjects with a reduction on TG levels were analyzed, a non-significant trend to increased Apo A–I levels (*p* = 0.099) and a significant increase in LCAT levels (*p* = 0.0397; Figure 4b) after the intake of ω3-milk was observed. These results highlight that the changes observed on the lipid-associated plasma protein fraction after the intake of ω3-milk are associated to the changes on the HDL proteomic profile.

### 3.2. Inflammation Associated Changes after PhyS-Milk Intake

**Figure 5:**
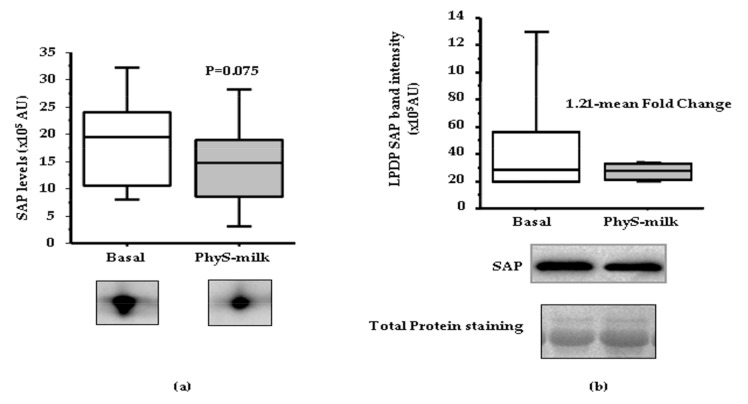
Serum amyloid P (SAP) proteomic profile. (**a**) Box-Plot diagram and 2-DE representative image showing a SAP decreasing trend in the soluble protein fraction of plasma (LPDP) proteomic profile after the intake of PhyS-milk (*p* = 0.075); (**b**) Box-Plot and representative western blot image showing changes in SAP in the LPDP samples after PhyS-milk intake (*N* = 5; 1.21-Fold change; *p* = 0.001).

Proteomic analysis of the lipoprotein-depleted-plasma (LPDP) fraction revealed that the intake of PhyS-milk induced a non-significant decreasing trend in the levels of the pro-inflammatory protein SAP when compared to the basal samples (*p* = 0.075; Figure 5a; Supplementary Table S3). This result was confirmed after Western blot (WB) validation where a 1.21-mean fold decrease in SAP levels was detected in LPDP samples after the intake of PhyS-milk (*p* = 0.001; Figure 5b). 

On the contrary, no changes were observed in the LPDP fraction after the intake of ω3-milk (Supplementary Table S4).

**Figure 6:**
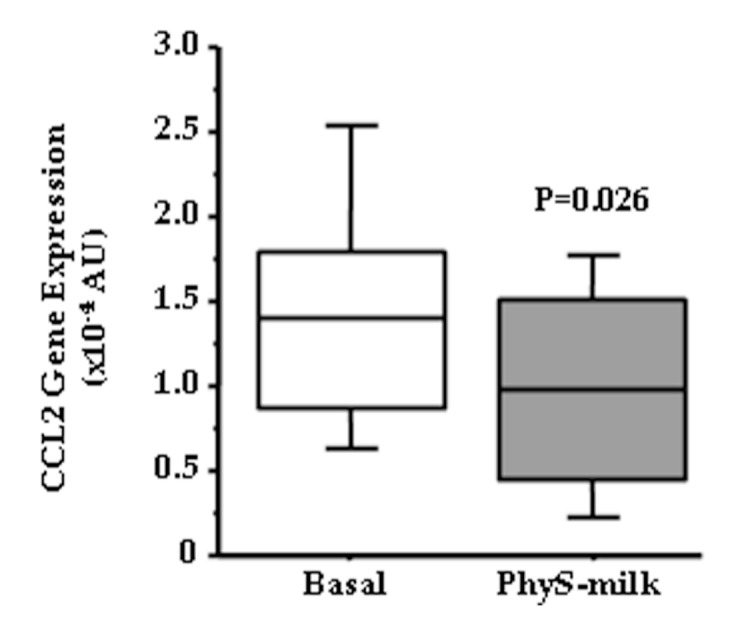
C-C motif chemokine 2 (CCL2) gene expression. Box-plot diagram showing the significant decrease in CCL2 gene expression after the intake of PhyS-milk in the peripheral blood leukocyte fraction of a randomly selected group of subjects (*N* = 12; *p* = 0.026).

Due to the observed changes in the inflammation-associated pentraxin SAP after the intake of PhyS-milk, we investigated if PhyS-milk could induce changes in the expression of the key pro-inflammatory chemokine in inflammatory cells, C-C motif chemokine 2 (CCL2). This analysis revealed a significant reduction of the CCL2 transcripts after the intake of PhyS-milk (*p* = 0.03; Figure 6). In addition, a trend towards increased expression levels of the Interleukin 10 receptor (IL-10R; *p* = 0.06) was also observed.

## 4. Discussion

Nutritional intervention is a useful strategy for the prevention of many diseases, especially in those in which obesity is a risk factor [27]. Phytosterols (PhyS) are natural components derived from plants and vegetable oils with a similar structure to cholesterol that are not synthesized in humans [28]. Through their ability to interfere with cholesterol metabolism, PhyS have been shown to effectively reduce cholesterol levels, and possibly beneficially affect CVD risk prevention [29].

Our group and others have demonstrated that the intake of plant stanols and sterols, in different food-platforms, reduces serum levels of total cholesterol. However, controversy exists regarding plant sterols-mediated protection as some studies have suggested a potential deleterious effect in relation to cardiovascular risk [30,31,32]. The explanation of these contradictory results seems to be related to the amount of stanol supplementation. In fact, it has been shown that the increase in stanol supplementation beyond the maximum recommendable dose of 3 mg/day is not associated with a higher reduction of cholesterol levels [33]. Importantly, in our study, an intake of 1.6 g PhyS/day (value within the recommended dose range) with low fat milk for four weeks reduced total cholesterol, non-HDL-C, and LDL-C, and also reduced the susceptibility of LDL to oxidation [7]. It is important to highlight, that in the present study, each patient is his/her own control as all the analyzed variables have been compared to the baseline levels measured in each subject after a wash-out run-in period with plain low-fat milk. Indeed, the aim of the clinical trial was to compare the effects of both PhyS- and ω3-milk interventions. Although we cannot exclude a potential effect inherent to the participation in the study, the two-arm crossover design with the two bioactive products minimizes the potential influence of this effect in the results. 

Here, we have observed a significant decrease in the pro-inflammatory pentraxin SAP in the soluble fraction of plasma (LPDP) of the healthy-overweight volunteers after the intake for four weeks of PhyS-milk. SAP plays an important role in innate immunity and in the atherosclerotic process [34]. Pentraxins have been detected within advanced human atherosclerotic plaques suggested to play an active role in atherogenesis [35]. Indeed, we have previously demonstrated an important increase in SAP levels in the early phase post acute myocardial infarction (AMI), and an even higher increase of this protein 3 days after the event [36]. SAP deficiency prevents atherosclerosis [37] and it is involved in other key biological processes for the cardiovascular system, such as inflammation, fibrosis, and coagulation [34,38]. Specifically, pentraxins have been shown to contribute to the chronic low-grade inflammatory state that characterizes obesity [39]. Therefore, the intake of PhyS-milk can reduce the cardiovascular risk associated to overweight and obesity by reducing the pro-inflammatory protein SAP.

Moreover, we have also found a significant decrease in the expression of the gene encoding for C-C motif chemokine 2 (CCL2), also known as monocyte chemotactic protein-1(MCP-1), and a trend to decreased the IL10-R, two important cytokines with pro- and anti-inflammatory properties respectively involved in CVD, after dietary intervention with PhyS-milk. In fact, it was shown that the intake of PhyS produces an anti-inflammatory reaction by acting on cytokines’ activity [40]. CCL2/MCP-1 is a key driver of adipose tissue inflammation in obesity [41]. Indeed, it is directly implicated in the propagation of the chronic low-grade inflammatory state associated to obesity [42,43,44] through its role as a monocyte attractant, which is the major cell that differentiates into macrophages and foam cells in the atherosclerotic lesion [40]. On the other hand, IL-10 is an anti-inflammatory protein by suppressing the synthesis of pro-inflammatory cytokines and also by inhibiting the activation of macrophages [45]. Therefore, our results highlight that PhyS-milk consumption may induce a shift towards a decreased inflammatory state through its effect on two key cytokines, CCL2/MCP-1 and IL-10, having thus a potential effect in the context of obesity where inflammatory pathways are exacerbated. 

The cardiovascular benefits of fish oils are well known and directly attributed to the effects of ω3 fatty acids (FA) [46]. Beneficial effects of ω3 and ω6 FA have been compared and the results suggest that higher levels of ω3 FA are protective against atherosclerosis. Therefore, modern western diets are directed towards a reduction of the ω6/ω3 FA ratio to protect against atherosclerosis [47,48]. Previous studies of our group have shown that the ingestion of this ω3-milk reduces the levels of triglycerides (TG) and palmitic acid [7]. Palmitic acid is a major component of the western diet and is associated with insulin resistance and glucose intolerance, becoming an important risk factor of diabetes and CVD [49]. Importantly, these metabolic alterations are often present in obese individuals. Our proteomic approach has shown that regular intake of ω3-milk results in a significant increase of Apo E protein level in LDL. It had been suggested that ω3 FA effects in reducing TG levels are dependent on Apo E [47]. Indeed, the beneficial effects of ω3 FA are partially blunted in Apo E deficient (Apo E^−/−^) mice, suggesting that Apo E is necessary for the cardioprotective effects exerted by ω3 [50]. Accordingly, diverse studies have shown that *Apo E*^−/−^ mice suffer hypercholesterolemia and an association between Apo E levels and CVD has also been reported in humans [51,52]. Even more, it has been shown that in the absence of some isoforms of Apo E, the beneficial cardioprotective effect of ω3 PUFA may be partially lost [53,54]. Of note, our results highlight an association between Apo E increase in LDL and an improved TG profile in obese subjects after four weeks intervention with ω3-milk. 

**Figure 7:**
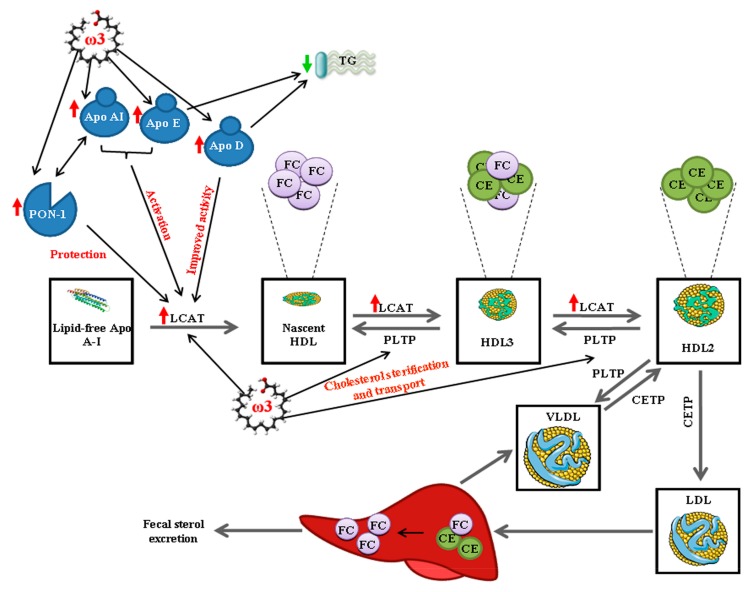
Simplified diagram of the lipid metabolism canonical pathway. Maturation of HDL and free cholesterol (FC) esterification are catalyzed by LCAT. Apo A-I and Apo E activate LCAT, and PON-I, which is also activated by Apo A–I, avoiding LCAT inactivation. Apo D binds to LCAT to improve its esterification activity. Finally, cholesterol is transported to the liver for its elimination by fecal excretion.

Importantly, herein we describe for the first time that the intake of ω3-milk induces a coordinated increase of several HDL associated proteins. Diverse studies show that the antioxidant effect of HDL is associated to a coordinated action of different proteins that allow HDL to protect LDL from oxidation for a longer period [18,55]. This correlates with the importance of HDL composition, emphasizing on HDL quality rather than HDL quantity. Apo A–I is the major protein component of HDL and therefore the cardioprotective benefits of HDL are often associated to this protein. High levels of Apo A–I are associated to a reduced vascular lesion and to an increase in the HDL plasma level [56]. It is widely known that Apo A–I is implicated on cholesterol transport from peripheral tissues to the liver for its clearance by the formation and stabilization of the HDL, but many additional proteins are also implicated in this process [9]. Lipid-free Apo A–I produces the nascent HDL particles and LCAT catalyzes HDL maturation by the esterification of free cholesterol (FC) into cholesteryl esters (CE), being both particles accumulated into the core of the HDL particle. Then, cholesterol ester transfer protein (CEPT) transfer CE particles for the mature form of HDL (HDL2) to VLDL and LDL to be finally excreted and eliminated [57,58]. LCAT is essential for cholesterol esterification, but also needs the presence of other proteins to be activated. Thus, Apo A–I and Apo E are the best LCAT activators in plasma and PON-1 protects LCAT from its inactivation [58]. PON-1 appears predominantly associated to HDL, especially to HDL3 and it is activated and stabilized by Apo A–I. PON-1 is an atheroprotective protein with an important role as an anti-oxidant [57]. Interestingly, in the present study we have found that ω3-milk intake significantly increases the HDL content of key proteins involved in HDL metabolism such as Apo A–I and LCAT. Those changes are specifically associated to a reduction in TG plasma levels after the intake of ω3-milk. Thus, it is conceivable that ω3 by increasing Apo A–I and LCAT levels enhances both cholesterol esterification and transport, significantly improving the lipoprotein profile (Figure 7). Furthermore, the ω3-milk-induced increase observed in PON1 could not only enhance LCAT activity but could also improve the anti-oxidant abilities of HDL particles. In addition, we observed an increase in Apo D HDL levels after the intake of ω3-milk. Apo D has been implicated in the reduction of TG plasma levels and also binds to LCAT improving its esterification activity [59]. Thus, the increase observed in Apo D after ω3-milk intake could contribute not only to the reduction in TG plasma levels but also to a decrease in VLDL-C levels (Figure 7). Furthermore, our study has shown that ω3-milk intake increases Apo L1 HDL content in overweight subjects. Apo L1 is mainly studied in chronic kidney disease, but studies in our group showed that a coordinated decrease in HDL content of both LCAT and Apo L1 seem to predispose to the presentation of acute ischemic events in hypercholesterolemia patients [24]. Therefore, the coordinated increase in both HDL-associated proteins after ω3-milk intake could reduce the CVD risk in high risk subjects such as those with overweight and metabolic syndrome.

## 5. Conclusions

In summary, our study supports the concept of supplementing diets with ω3 FA and PhyS to prevent CVD by inducing a coordinated change of the lipid-associated plasma protein fraction profile and by reducing inflammation. These changes are then translated into a significant improvement of the lipid profile. This is of great importance in obese subjects, since they are more predisposed to suffer metabolic alterations and therefore increased risk of CVD. A healthy lifestyle and appropriate nutrition are key factors in the prevention of CVD, and the results of the present study support the notion that diet supplementation with PhyS and ω3 FA has beneficial effects against CVD in overweight and obese individuals by acting on two hallmarks of CVD progression, inflammation, and lipid metabolism.

## Figures and Tables

**Table 1:** Demographic and biochemical profile.

Product	PhyS-Milk		ω3-Milk	
Women/Men	19/13		19/13	
Age (Years)	50.5 ± 1.6		50.5 ± 1.6	
Parameter	Baseline	After PhyS-milk	Baseline	After ω3-milk
BMI	28.2 ± 0.7	28.1 ± 0.7	28.3 ± 0.7	28.2 ± 0.7
Ch (mg/dL)	216.0 ± 6.0	204.5 ± 5.6 *	216.4 ± 6.1	213.78 ± 5.9
TG (mg/dL)	110.2 ± 10.3	115.2 ±15.1	116.3 ± 14.3	99.5 ± 8.7 *
HDL-C (mg/dL)	54.5 ± 3.1	54.5 ± 3.0	57.0 ± 2.9	56.4 ± 2.8
LDL-C (mg/dL)	137.7 ± 4.9	127.2 ± 4.7 *	136.4 ± 5.0	137.7 ± 5.0
VLDL-C (mg/dL)	22.0 ± 2.0	23.0 ± 3.0	23.2 ± 2.9	19.8 ± 1.7 *
Non-HDL-C (mg/dL)	159.5 ± 5.8	150.0 ± 5.8 *	159.4 ± 6.0	157.3 ± 6.0

* Significant decrease after the intake of PhyS/ω3-milk (*p* < 0.05); Data are given as mean ± SEM. BMI = body mass index; Ch = cholesterol; HDL = high density lipoproteins; LDL = low density cholesterol; PhyS = phytosterols; TG = triglyceride; VLDL = very-low density lipoproteins.

## Supplementary Materials

The following are available online at www.mdpi.com/2072-6643/9/6/599/s1, Figure S1: In silico analysis of HDL protein changes, Table S1: Lipid and inflammation proteins, Table S2: Lipid and inflammation proteins, Table S3: Protein composition in lipoprotein depleted plasma after PhyS-milk intake, Table S4: Protein composition in lipoprotein depleted plasma after ω3-milk intake.
