# Supplementary material for: Phytosterols and Omega 3 Supplementation Exert Novel Regulatory Effects on Metabolic and Inflammatory Pathways: A Proteomic Study
Source: Nutrients. 2017 Jun 13;9(6):599. doi: 10.3390/nu9060599 (PMC5490578; doi:10.3390/nu9060599)
Supplement: Supplementary file 1 [file nutrients-09-00599-s001.zip › nutrients-194804-supplementary.pdf]

Supplemental Table S1. **Lipid and inflammation proteins.** Average protein composition after 4-weeks intervention with low-fat milk supplemented with Phytosterols

	Protein	Basal*	PhyS*	PhyS/B	*P-value*
LDL	Apo A-I	30.5	26.7	0.79	0.22
	Apo A-IV	1.4	1.9	1.64	0.08
	Apo CIII	6	3.7	0.43	0.18
	Apo D	8.1	4.4	0.60	0.14
	Apo E	23.5	25.6	0.96	0.89
	Apo J	2.2	2.4	0.92	0.50
	Apo L1	0.7	0.7	1.30	0.22
HDL	AAT	2.46	2.4616	1.00	0.99
	Apo D	8.80	9.11	1.04	0.55
	Apo L1	3.17	2.69	0.85	0.13
	Apo M	1.47	1.79	1.21	0.12
	Apo AIV	1.39	1.34	0.96	0.59
	Apo AI	1038.53	799	0.77	0.46
	Apo E	6.24	6.05	0.97	0.72
	C3 Complement	0.07	0.07	0.92	0.69
	Fibrinogen gamma chain	0.02	0.02	0.93	0.50
	HPT	0.26	0.26	0.99	0.95
	LCAT	0.81	0.94	1.17	0.22
	PON-1	3.80	3.52	0.92	0.18
	Serum albumin	7.56	8.01	1.06	0.64
	Serum Amyloid A-4	0.28	0.39	1.39	0.08
	TTR	0.20	0.25	1.20	0.12

* Protein intensity corrected by total-cholesterol levels, expressed as mean value of all subjects x10^5 AU

AAT=alpha-1 antitrypsin; Apo=apolipoprotein; HDL=high density lipoproteins; HPT=haptoglobin; LCAT=lecitin cholesterol acyltransferase; LDL=low density lipoproteins; PhyS=phytosterols; PON-1=paraoxonase-1; TTR=transthyretin.

Supplemental Table S2. Lipid and inflammation proteins. Average protein composition after 4-weeks intervention with low-fat milk supplemented with Omega 3

	Protein	Basal*	ω3*	ω3/B	*P*-value
LDL	Apo A-I	105.4	170.5	1.8	0.50
	Apo A-IV	7.3	5.9	0.83	0.23
	Apo CIII	44.1	29.2	0.63	-
	Apo D	20.7	35.3	1.3	0.14

	**Apo E**	120.8	138.9	1.5	**0.04**
	**Apo J**	11.9	21.5	1.7	0.14
	**Apo L1**	2.7	2.8	1.00	0.89
**HDL**	**AAT**	12.19	17.64	1.45	0.13
	**Apo D**	40.07	45.29	1.13	**0.008**
	**Apo L1**	11.30	16.54	1.46	**0.04**
	**Apo M**	6.99	7.58	1.08	0.23
	**Apo AIV**	7.29	8.22	1.13	0.22
	**Apo AI**	3577.60	4733.38	1.32	**0.009**
	**Apo E**	32.48	42.26	1.30	0.22
	**C3 Complement**	0.35	1.03	2.95	0.25
	**Fibrinogen gamma chain**	1.30	2.12	1.63	0.28
	**HPT**	3.79	4.79	1.26	0.15
	**LCAT**	19.24	22.40	1.16	**0.04**
	**PON-1**	35.23	49.10	1.39	**0.05**
	**Serumalbumin**	2.58	1.39	0.54	0.24
	**SerumAmyloid A-4**	0.98	1.56	1.60	0.24
	**TTR**	0.07	0.47	6.35	0.11

* Protein intensity corrected by triglyceride levels, expressed as mean value of all subjects x10^5 AU

AAT=alpha-1 antitrypsin; Apo=apolipoprotein; HDL=high density lipoproteins; HPT=haptoglobin; LCAT=lecitin cholesterol acyltransferase; LDL=low density lipoproteins; PON-1=paraoxonase-1; TTR=transthyretin.

Supplemental Table S3. Protein composition in lipoprotein depleted plasma after PhyS-milk intake.

Protname	Basal*	PhyS*	PhyS/B	*P* Value
**Alpha-1-antitripsin**	5.47	14.57	2.66	0.18
**Alpha-1B-glycoprotein**	260.46	233.79	1.11	0.47
**Alpha-2-antiplasmin**	40.03	33.43	0.83	0.14
**Apo A-I**	2.81	9.18	3.27	0.14
**Apo A-IV**	122.39	129.95	1.06	0.72
**Apo E**	16.46	15.34	0.93	0.47
**Apo J**	181.43	176.98	0.98	0.47
**Beta-2-glycoprotein 1**	48.15	63.03	0.76	0.47
**Complement C1r**	8.81	4.9	0.56	0.47
**Complement factor I**	6.15	10.02	1.63	0.47
**Fetuin A**	555.66	482.87	1.15	0.47
**Hemopexin**	1069.61	1033.66	0.97	0.47
**HPT**	218.92	147.46	1.48	0.72
**Kininogen**	5.27	4.37	1.20	0.07
**mTTR**	49.71	55.35	1.11	0.47
**Protein AMBP**	18.02	18.28	1.01	0.47

**RBP4**	131.19	106.29	1.23	0.47
**SAP**	15.08	11.03	0.73	0.07
**Serumalbumin**	24.55	20.23	1.21	1.00
**Vitronectin**	24.21	16.29	0.67	0.72
**Zinc-alpha-2-glycoprotein**	66.74	72.36	1.08	1.00

* Protein intensity, expressed as mean value of all subjects x10^5 AU

AMBP=alpha-1-microglobulin/bikunin precursor; Apo=apolipoprotein; HPT=haptoglobin; PhyS=phytosterols; mTTR=transthyretin monomer; RBP4=retinol binding protein 4; SAP=serum amyloid P.

Supplemental Table S4. Protein composition in lipoprotein depleted plasma after ω3-milk intake.

Protname	Basal	ω3	ω3/B	*P* Value
**Alpha-1-antitripsin**	2.16	2.83	0.76	0.59
**Alpha-1B-glycoprotein**	243.38	304.44	0.80	0.72
**Alpha-2-antiplasmin**	34.94	34.38	0.98	0.72
**Apo A-I**	3.17	4.66	0.68	0.11
**Apo A-IV**	133.11	150.49	1.13	1.00
**Apo E**	23.31	22.98	0.99	0.27
**Apo J Total**	213.17	215.08	1.01	1.00
**Beta-2-glycoprotein 1**	58	55.25	0.95	0.72
**Complement C1r**	12.33	12.29	1.00	0.18
**Complement factor I**	6.99	7.02	1.00	0.72
**Fetuin A**	490.18	549.62	1.12	0.47
**Hemopexin**	897.6	1184.26	0.76	0.14
**HPT**	178.94	200.2	1.12	0.07
**Kininogen**	4.63	6.68	0.69	0.47
**mTTR**	58.14	64.96	1.12	1.00
**Protein AMBP**	24.17	21.1	1.15	0.07
**RBP4**	118.88	142.04	0.83	0.14
**SAP**	18.99	17.17	0.90	0.47
**Serumalbumin**	27.02	24.69	0.91	0.07
**Vitronectin**	25.09	21.37	1.17	0.72
**Zinc-alpha-2-glycoprotein**	61.02	72.09	1.18	0.47

* Protein intensity, expressed as mean value of all subjects x10^5 AU

AMBP=alpha-1-microglobulin/bikunin precursor; Apo=apolipoprotein; HPT=haptoglobin; mTTR=transthyretin monomer; RBP4=retinol binding protein 4; SAP=serum amyloid P.

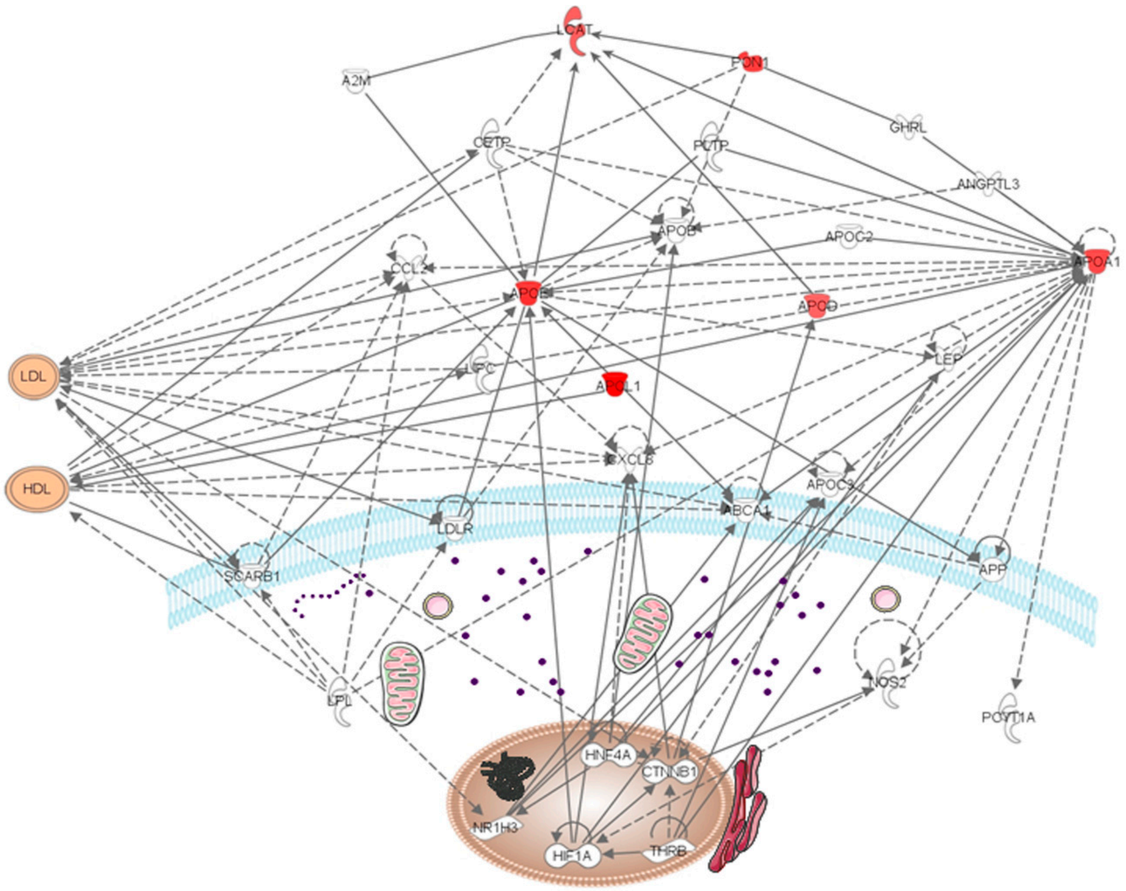

Supplemental Figure S1. ***In silico*** **analysis of HDL protein changes.** Bioinformatic analysis using the Ingenuity Systems Pathway Analysis software showing specific changes in the Lipid Metabolism neural network.
